# The association between preventive health and outpatient spending and life expectancy by income quartile

**DOI:** 10.1186/s12939-022-01748-8

**Published:** 2022-11-12

**Authors:** Mason S. Barnard, Rama M. Hagos

**Affiliations:** 1 Present Address: Department of Sociology, Princeton University, Wallace Hall, Princeton, NJ 08540 USA; 2 Office of Population Research, Princeton University, Wallace Hall, Princeton, NJ 08540 USA

**Keywords:** Preventive care, Health access, Health quality, Longevity

## Abstract

**Background:**

To describe the relationship between longevity and local access to preventive healthcare at the county level.

**Methods:**

We used Medicare outpatient reimbursement data from the 2010 Dartmouth Health Atlas and longevity data from Chetty et al. (2016) to identify the cross-sectional associations between county longevity, access to outpatient care, and the quality of primary care.

**Results:**

We find that the cost of outpatient care is inversely correlated with area life expectancy for individuals in the bottom income quartile. Much of this correlation is driven by men in the bottom income quartile. We also find that disaggregating a preventive care index produces significant relationships between components of the index and longevity where none were previously found.

**Conclusions:**

These results counter prior assertions that local health costs are not associated with life expectancy. Additionally, the results also suggest that the local cost of outpatient care and the quality of that care may influence the longevity of low-income populations, especially for low-income men.

## Background

Although well-studied, the relationship between health and healthcare spending is often simplified into a relationship between longevity and dollars spent. Chetty et al.’s (2016) landmark paper uses overall Medicare reimbursements as a proxy for health access, finding no relationship between a county’s average reimbursements and life expectancy [1]. Regional comparisons across the United States likewise suggest that more health care spending shows little association with better health outcomes [2, 3]. Medicare patients in regions with high spending use more healthcare resources but do not see any boosts to longevity or other health outcomes [2]. Similar studies suggest that among developed countries, higher overall health spending is not significantly associated with health outcomes [4].

Yet, dollars spent in different healthcare settings are not equivalent. Abundant evidence suggests that spending on preventive and maintenance care improves overall longevity and health [5], while spending on inpatient and elderly care often outweighs potential health benefits [6, 7]. Prior authors observe such a relationship in Chetty et al. (2016)’s initial results. Referencing the paper’s supplementary analysis, one commentary notes that fewer discharges for ambulatory-sensitive conditions, a key measure of primary care access, were correlated with longer life expectancy [8]. The variations in the value of spending by care type make implicit sense. Costly procedures at end-of-life may prolong life expectancy by just a few months, if at all, while regular checkups, kidney dialysis, cholesterol medications, and good exercise habits extend life spans by decades. The aggregated preventive care metrics may conceal potentially significant data and bias it towards certain types of care, such as diabetes—the subject of three of the six metrics used in the index. By lumping costly inpatient procedures and end of life care with cheaper, more impactful outpatient visits and surgeries, metrics of overall spending may not adequately represent the relationship between spending and health [9]. These potential issues call for more investigation into the relationship between health access and longevity.

Quality of care indices may similarly obscure the ties between different types of care and longevity. Evidence connecting quality measures to better outcomes remains modest [10], with the association between care quality and health outcomes often dependent on the specific measure used and the population of patients assessed [11]. Prior studies that show little or weak associations between preventive care quality and health outcomes also tend to rely on an index of such measures [1, 12]. Such aggregation, while useful, can mask significant relationships between different dimensions of care quality and health.

Given the limitations of composite health metrics, this paper assesses the cross-sectional relationship between longevity and county-level preventive care spending and quality. It draws from the methodology and data originally used in Chetty et al. (2016) and nuances our understanding of the relationship between health, healthcare spending, and healthcare quality by focusing on outpatient reimbursements and preventive care.

## Methods

To estimate local life expectancy, we obtained the aggregate data files that the Health Inequality Project team built from IRS tax records and SSA death records from 1999 to 2014. Using the former paper’s source code, we first reproduced estimates of period life expectancy at age 40 for men and women by income quartile for every county in the country. For individuals older than 76, Gompertz parameters were used to extrapolate mortality rates and derive life expectancies. Finally, all life expectancy estimates were adjusted to control for county-level racial and ethnic composition.

To estimate the cost of preventive care, we obtained county-level data for all Medicare outpatient reimbursements from the Dartmouth Atlas of Health Care (DAHC) for 2010. We used the DAHC’s disaggregated primary care index to create six individual metrics: the percentage of individuals with a PCP visit, the percentage of women over 67 with a mammogram, the percentage of diabetics with a hemoglobin test, the percentage of diabetics with an eye exam, the percentage of diabetics with a lipid test, and the discharge rate for ambulatory-sensitive conditions, such as chronic obstructive pulmonary disease (COPD) and asthma [13]. A full list of ambulatory sensitive conditions considered in this paper is in Appendix A.

We then calculated Pearson correlation measures between Medicare outpatient reimbursements, the disaggregated primary care index, and county life expectancy at the bottom and top income quartiles, weighted by county population. As a sensitivity analysis, we used the Benjamini–Hochberg procedure to correct for multiple hypothesis testing and to decrease the false discovery rate. These results are reported in Appendix B. We also conducted analyses for both male and female life expectancy and for pooled life expectancy. Finally, we ran analyses for the second and third income quartiles as supplementary analyses. All analyses were performed using Stata version 16 (StataCorp, College Station, TX).

## Results

**Fig. 1:**
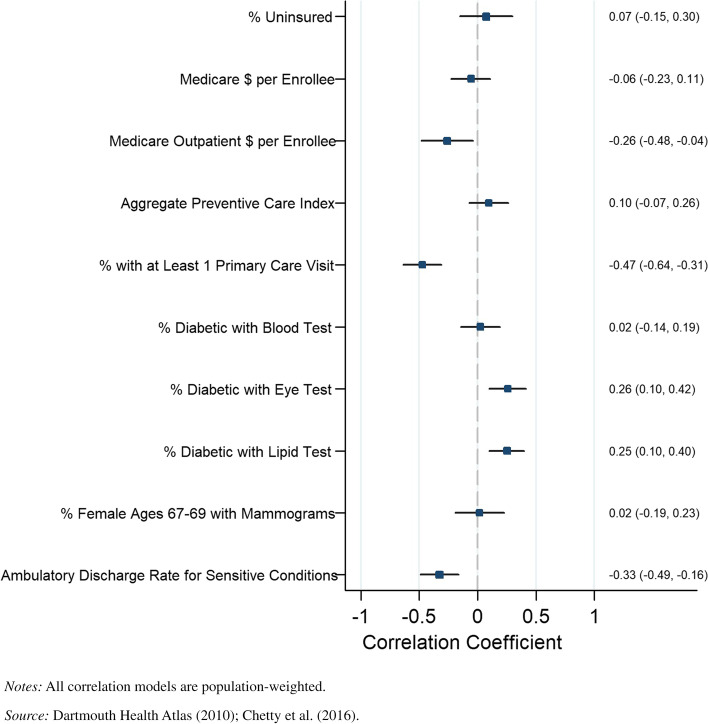
County-level Pearson correlations between preventive health spending and quality measures and life expectancy, bottom income quartile

Our primary results for those in the bottom income quartile are displayed in Fig. 1. Life expectancy was negatively correlated with outpatient Medicare reimbursements for the bottom income quartiles in counties (*r* = -0.26, *P* = 0.025). Among our disaggregated preventive care metrics, the percentage of diabetics receiving eye exams (*r* = 0.26, *P* = 0.002) and the percentage of diabetics receiving lipid tests (*r* = 0.25, *P* = 0.002) were positively associated with life expectancy in the bottom quartile at the county level. The discharge rate for ambulatory-sensitive conditions was negatively associated with life expectancy (*r* = -0.33, *P* = 0.000). The percentage of individuals receiving a primary care visit was also negatively correlated with longevity (*r* = -0.47, *P* = 0.000) among individuals in the bottom income quartile. Finally, the percentage of females receiving a mammogram and the percentage of diabetics receiving blood tests both bore no statistically significant relationship with longevity among individuals in the bottom income quartile.

**Fig. 2:**
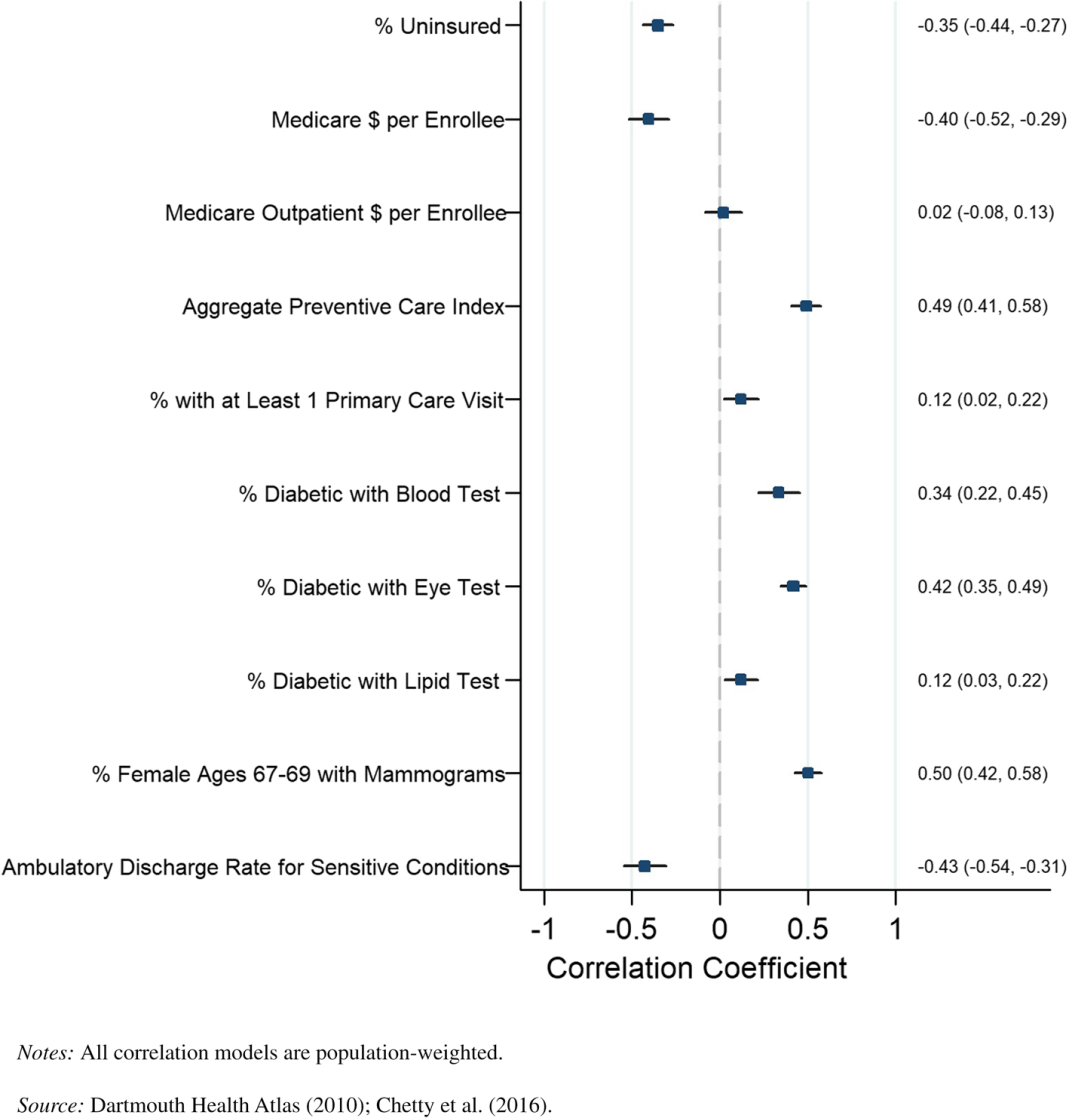
County-level Pearson correlations between preventive health spending and quality measures and life expectancy, top income quartile

In comparison, for individuals in the top quartile, four out of six preventive care metrics were positively associated with life expectancy, as seen in Fig. 2. Among our disaggregated preventive care metrics, the percentage of diabetics receiving blood tests (*r* = 0.34, *P* = 0.000), the percentage of diabetics receiving eye exams (*r* = 0.42, *P* = 0.000), the percentage of diabetics receiving lipid tests (*r* = 0.12, *P* = 0.013), and the percentage of individuals receiving a primary care visit (*r* = 0.12, *P* = 0.017) were positively correlated with longevity among individuals in the top income quartile at the county level. The discharge rate for ambulatory-sensitive conditions (*r* = -0.43, *P* = 0.000) and the percentage of females receiving a mammogram (*r* = 0.50, *P* = 0.000) were both negatively correlated with longevity among individuals in the top income quartile. Life expectancy displayed no statistically significant association with outpatient Medicare reimbursements for the top income quartiles.

We conducted supplementary analyses for the second and third income quartiles and found similar patterns to the top income quartile. These results are reported in Appendix C.

**Fig. 3:**
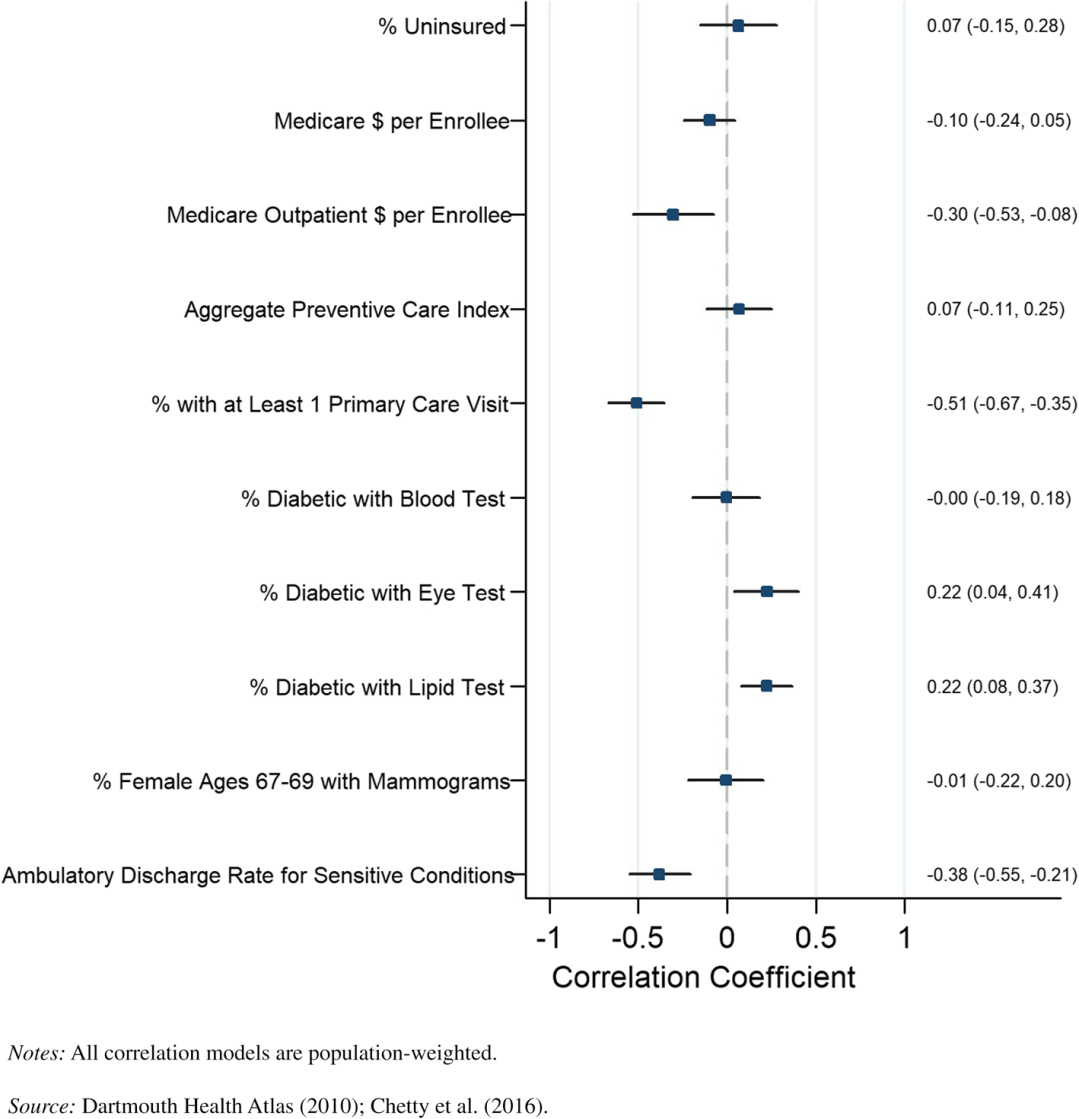
County-level Pearson correlations between preventive health spending and quality measures and life expectancy, men in bottom income quartile

**Fig. 4:**
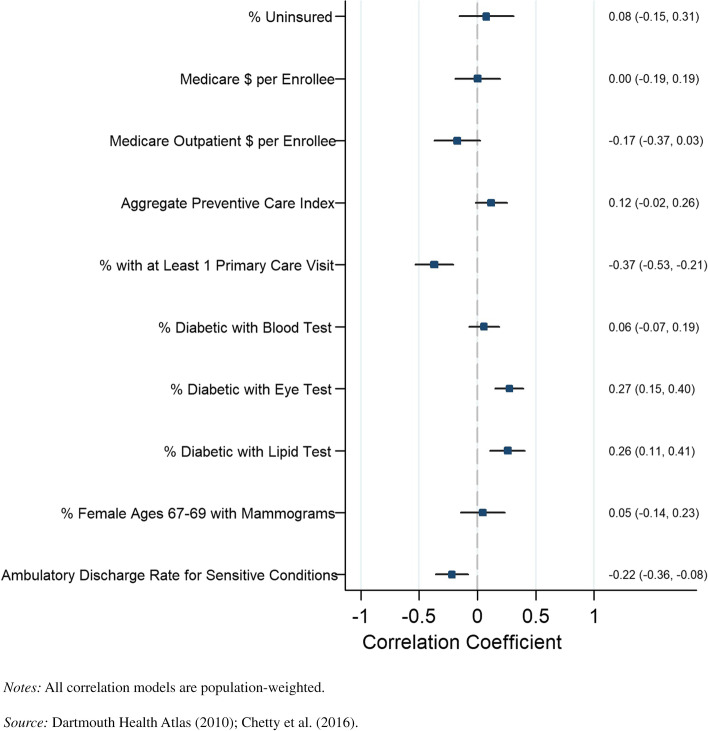
County-level Pearson correlations between preventive health spending and quality measures and life expectancy, women in bottom income quartile. *Notes:* All correlation models are population-weighted. *Source:* Dartmouth Health Atlas (2010); Chetty et al. (2016)

We also looked at the association between our disaggregated preventive care metrics and life expectancy by income quartile and sex at the county level. The results for the bottom income quartile for men are presented in Fig. 3, and the results for women in the bottom quartile are presented in Fig. 4. Similar patterns were identified by sex when looking at the percentage of diabetics receiving eye exams, percentage of diabetics receiving lipid tests, percentage of diabetics receiving blood tests, and the discharge rate for ambulatory-sensitive conditions. The percentage of individuals receiving a primary care visit had a greater negative correlation for men (*r* =  − 0.51, *P* = 0.000) than women (*r* =  − 0.37, *P* = 0.000). Life expectancy was negatively correlated and statistically significant with outpatient Medicare reimbursement for men in the bottom income quartiles in counties (*r* =  − 0.30, *P* = 0.000), but this result was not robust for females in the bottom income quartiles.

**Fig. 5:**
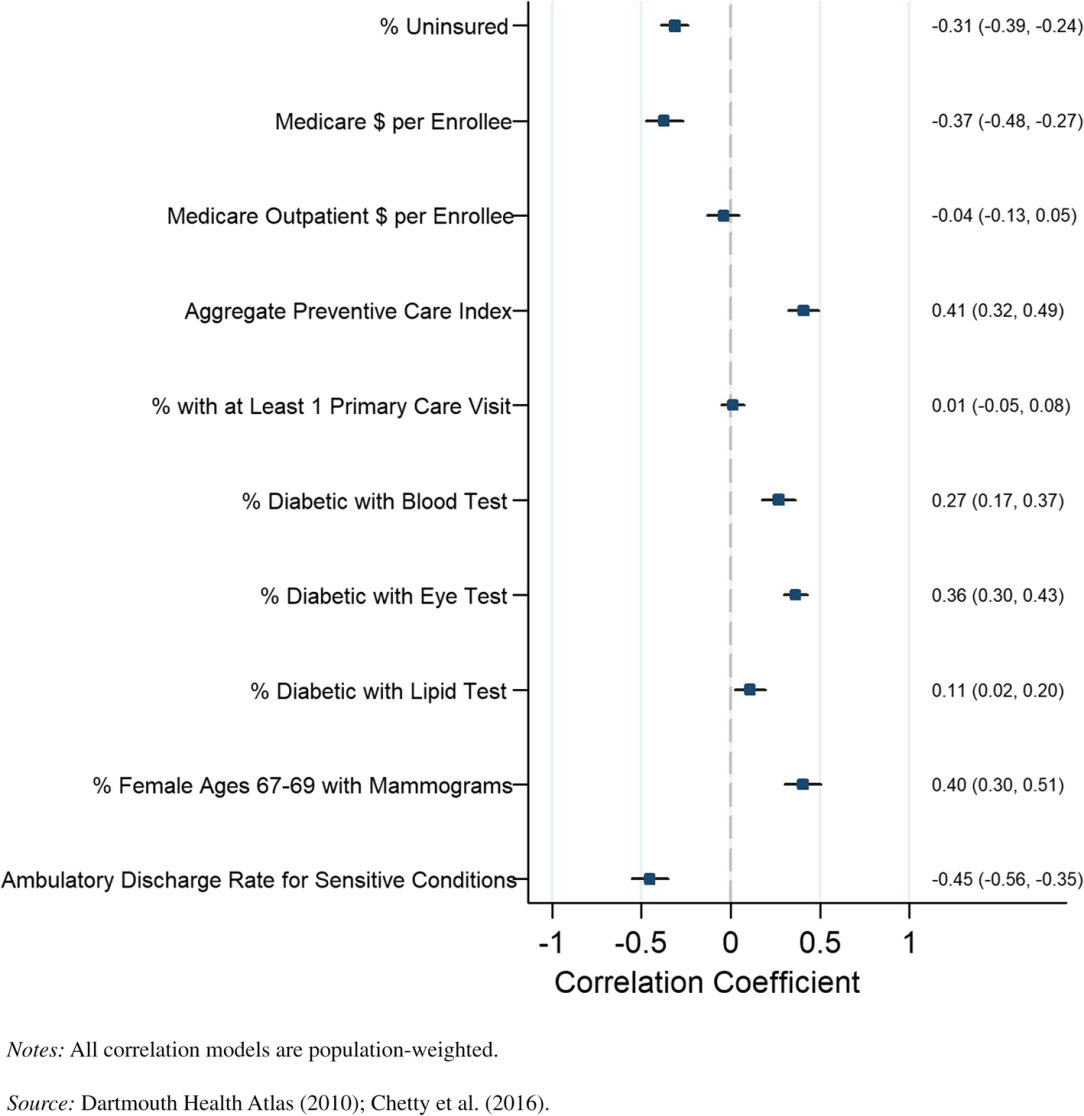
County-level Pearson correlations between preventive health spending and quality measures and life expectancy, men in top income quartile

**Fig. 6:**
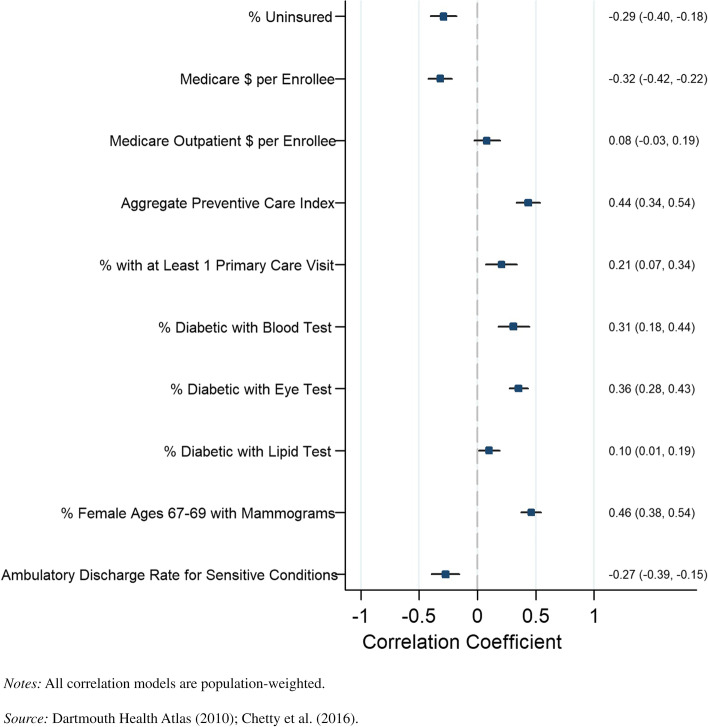
County-level Pearson correlations between preventive health spending and quality measures and life expectancy, women in top income quartile

The results for the top income quartile by sex are presented in Fig. 5 for men and Fig. 6 for women, respectively. Similar patterns were identified by sex when looking at the percentage of diabetics receiving eye exams, percentage of diabetics receiving lipid tests, percentage of diabetics receiving blood tests, and discharge rate for ambulatory-sensitive conditions. The percentage of individuals receiving a primary care visit had a positive and statistically significant correlation for women (*r* = 0.21, *P* = 0.003), but this relationship was not significant for men. Life expectancy was not significantly correlated with outpatient Medicare reimbursements for men and women in the top income quartiles in counties.

## Discussion

Our results suggest that there is a strong, negative association at the county level between the cost of outpatient care and life expectancy for individuals in the bottom income quartile. Interestingly, we found that this association is primarily driven by men in the bottom income quartile. Previous research has shown that low-income men have poorer health outcomes and are less likely to receive health care [14, 15]. This pattern may be the result of low-income individuals rationing elective care to avoid the cost, and is reinforced by the negative association between the ambulatory-sensitive condition discharge rate and life expectancy for those in the bottom income quartile. Rationing care among low-income men may save money in the short-term, but likely escalates both costs and mortality over time—an important finding for policymakers.

Our results further suggest that certain aspects of preventive health are significantly associated with longevity. But the nature of these associations vary. Among low-income individuals, two of our diabetes quality metrics display a positive association, indicating that quality of preventive care is linked to life expectancy, while our third metric, the percentage of individuals receiving a primary care visit, shows a negative association. The latter finding is surprising, as we would expect that seeing a doctor is associated with better health. However, it may be explained by self-selection, with healthier individuals less likely to see a physician. Among high-income individuals, all preventive care metrics were positively associated with life expectancy. This finding reinforces the connection between preventive care and health. Moreover, the consistent positive relationship between these metrics, particularly the percentage of individuals receiving a primary care visit, stands in stark contrast to the results seen among the bottom quartile of the income distribution. This contrast may suggest that while lower income individuals self-select into preventive care based on underlying health, wealthier individuals do not—lending a negative association with longevity for the former and a positive association for the latter.

## Conclusion

Past research has found mixed results on the relationship between health spending and life expectancy. We highlight findings that show that Medicare outpatient spending and some preventative care metrics are a better approximation of the costs and quality of health maintenance and are statistically significant with life expectancy at the county level. The importance of health spending is especially crucial in the midst of the Covid-19 pandemic, where differential access to care both before and during the pandemic has impacted the health of disadvantaged communities.

## Limitations

As we based the methodology of this paper on Chetty et al. (2016), we acknowledge the same limitations of the original paper. Life expectancy estimates were based on extrapolations of mortality rates after age 76 and age 63 for year-specific estimates. Income for individuals ages 63 or older was measured at age 61 because of previous evidence that earnings after age 61 are less correlated with earlier earnings due to the rate of retirement. All observations with an income of $0 were excluded from the analyses because they could not be tracked in the Social Security Administration death records data. Race and ethnicity information is not included in the IRS income data we used to estimate life expectancy by income and gender, so we can only adjust for county racial composition and cannot directly measure differences in life expectancies by race and ethnicity. Although this paper uses longitudinal data to estimate life expectancy, it assesses the local relationship between outpatient spending/quality and life expectancy on a cross-sectional basis. Future work should incorporate more data and build on our results with a longitudinal design.

## Figures and Tables

**Fig. 7:**
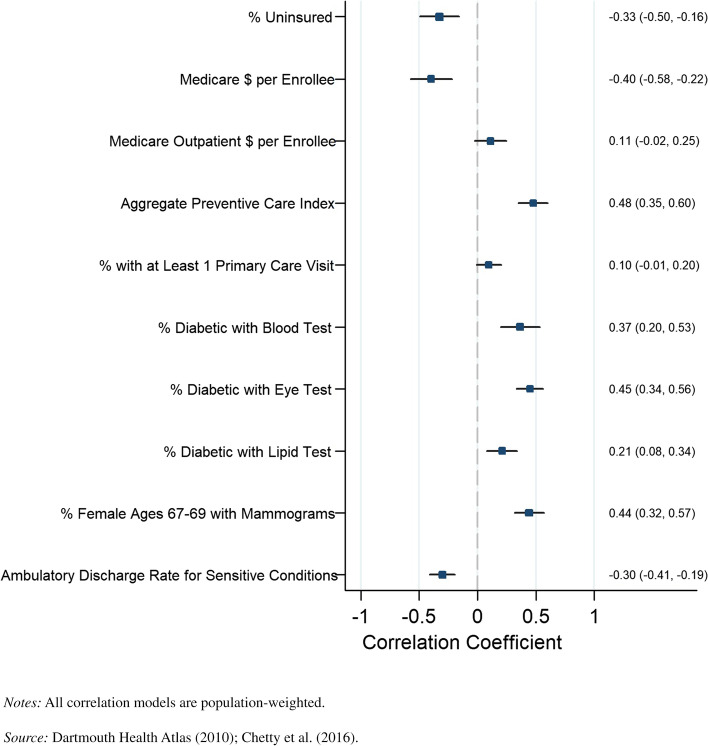
County-Level Pearson Correlations between Preventive Health Spending and Quality Measures and Life Expectancy, Second Income Quartile

**Fig. 8:**
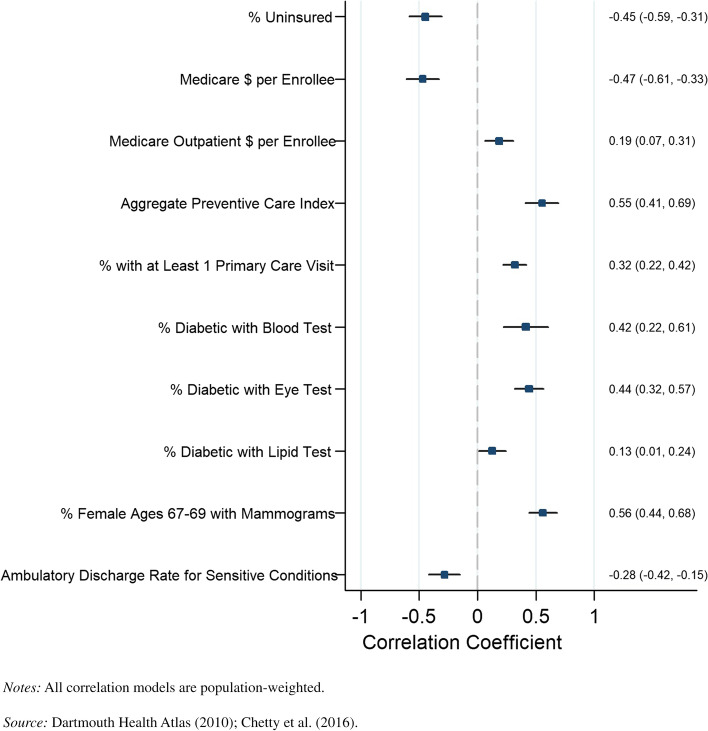
County-Level Pearson Correlations between Preventive Health Spending and Quality Measures and Life Expectancy, Third Income Quartile

## Data Availability

The datasets supporting the conclusions of this article are available on the Health Inequality Project website, https://healthinequality.org/data/ and the Dartmouth Health Atlas Project, https://data.dartmouthatlas.org/.

## Appendix A

### Ambulatory Care-Sensitive (ACS) Conditions

1. Convulsions (780.3x)
2. Chronic Obstructive Pulmonary Disease (COPD) (491xx, 492xx, 494xx, 496xx, 466.0x: 466.0x only w/secondary dx 491xx, 492xx, 494xx, 496xx)
3. Bacterial Pneumonia (481xx, 482.2x, 482.3x, 482.9x, 483xx, 485xx, 486xx: excl. secondary dx 282.6x)
4. Asthma (493xx)
5. Congestive Heart Failure (CHF) (428xx, 402.01, 402.11, 402.91, 518.4x: excl. sx 36.01, 36.02, 36.05, 36.1x,37.5x, or 37.7x)
6. Hypertension (401.0x, 401.9x, 402.00, 402.10, 402.90: excl. sx 36.01, 36.02, 36.05, 36.1x,37.5x, or 37.7x)
7. Angina (411.1x, 411.8x, 413xx: excl. sx 01-86.99)
8. Cellulitis (681xx, 682xx, 683xx, 686xx: excl. sx 01-86.99, unless 86.0x is the first and only sx code)
9. Diabetes (250.0x, 250.1x, 250.2x, 250.3x, 250.8x, 250.9x)
10. Gastroenteritis (558.9x)
11. Kidney/Urinary Infection (590xx, 599.0x, 599.9x)
12. Dehydration (276.5x).

Numerator counts are based on ICD-9-CM diagnosis codes. Surgical codes are usually excluded to ensure that the admission was for a medical condition.

## Appendix B

### Benjamini-Hochberg procedure *P*-values

**Table:**

**Q1 Variables**	***P*****-Values**	**Adjusted Value**
diabetes_test	0.782	0.050
diabetes_eye	0.002	0.020
mammogram_test	0.869	0.100
ambulatory_discharge	0.000	0.017
diabetes_lipids	0.002	0.025
vision_test	0.000	0.014
med_outpatient	0.025	0.033
**Q4 Variables**	***P*****-Values**	**Adjusted Value**
diabetes_test	0.000	0.014
diabetes_eye	0.000	0.017
mammogram_test	0.000	0.020
ambulatory_discharge	0.000	0.025
diabetes_lipids	0.013	0.033
vision_test	0.017	0.050
med_outpatient	0.687	0.100
