# Correction: The association between preventive health and outpatient spending and life expectancy by income quartile

**DOI:** 10.1186/s12939-022-01800-7

**Published:** 2023-01-13

**Authors:** Mason S. Barnard, Rama M. Hagos

**Affiliations:** 1 Present address: Department of Sociology, Princeton University, Wallace Hall, Princeton, NJ 08540 USA; 2 Office of Population Research, Princeton University, Wallace Hall, Princeton, NJ 08540 USA

**Correction: Int J Equity Health 21, 159 (2022)**

**https://doi.org/10.1186/s12939-022-01748-8**

After publication of this article [1], the authors reported that in the Funding information section the following was missing: 'This publication was supported by the Princeton University Library Open Access Fund.'

The original article [1] has been corrected.
